# Incidence and predictors of hospitalization in patients with atrial fibrillation: results from the Chinese atrial fibrillation registry study

**DOI:** 10.1186/s12872-021-01951-5

**Published:** 2021-03-19

**Authors:** Zhaojie Dong, Xin Du, Shangxin Lu, Chao Jiang, Shijun Xia, Liu He, Xin Su, Zhaoxu Jia, Deyong Long, Caihua Sang, Ribo Tang, Nian Liu, Rong Bai, Ronghui Yu, Jianzeng Dong, Changsheng Ma

**Affiliations:** 1 Department of Cardiology, Beijing Anzhen Hospital, Capital Medical University, National Clinical Research Centre for Cardiovascular Diseases, No. 2 Beijing Anzhen Road, Chaoyang District, Beijing, 100029 People’s Republic of China; 2 Heart Health Research Center, Beijing, People’s Republic of China; 3 The George Inst itute for Global Health, Faculty of Medicine, University of New South Wales, Sydney, Australia

**Keywords:** Hospitalization, Atrial fibrillation, Incidence, Predictors

## Abstract

**Background:**

Patients with atrial fibrillation (AF) underwent a high risk of hospitalization, which has not been paid much attention to in practice. Therefore, we aimed to assess the incidence, causes and predictors of hospitalization in AF patients.

**Methods:**

From August 2011 to December 2017, a total number of 20,172 AF patients from the Chinese Atrial Fibrillation Registry (China-AF) Study were prospectively selected for this study. We described the incidence, causes of hospitalization by age groups and sex. The Fine-Gray competing risk model was employed to identify predictors of first all-cause and first cause-specific hospitalization.

**Results:**

After a mean follow-up of 37.3 ± 20.4 months, 7,512 (37.2%) AF patients experienced one or more hospitalizations. The overall incidence of all-cause hospitalization was 24.0 per 100 patient-years. Patients aged < 65 years were predominantly hospitalized for AF (42.1% of the total hospitalizations); while patients aged 65–74 and ≥ 75 years were mainly hospitalized for non-cardiovascular diseases (43.6% and 49.3%, respectively). We found patients complicated with heart failure (HF)[hazard ratio (HR) 1.10, 95% confidence interval (CI) 1.02–1.18], established coronary artery disease (CAD) (HR 1.24, 95%CI 1.17–1.33), ischemic stroke/transient ischemic attack (TIA) (HR 1.22, 95%CI 1.15–1.30), diabetes (HR 1.14, 95%CI 1.08–1.20), chronic obstructive pulmonary disease (COPD) (HR 1.28, 95%CI 1.02–1.62), gastrointestinal disorder (HR 1.37, 95%CI 1.21–1.55), and renal dysfunction (HR 1.24, 95%CI 1.09–1.42) had higher risks of hospitalization.

**Conclusions:**

More than one-third of AF patients included in this study were hospitalized at least once during over 3-year follow-up. The main cause for hospitalization among the elderly patients (≥ 65 years) is non-cardiovascular diseases rather than AF. Multidisciplinary management of comorbidities should be advocated to reduce hospitalization in AF patients older than 65 years old.

*Clinical Registry*http://www.chictr.org.cn/showproj.aspx?proj=5831. Unique identifier: ChiCTR-OCH-13003729. The registration date is October 22, 2013.

**Supplementary Information:**

The online version contains supplementary material available at 10.1186/s12872-021-01951-5.

## Background

Atrial fibrillation (AF), considered as a global cardiovascular condition, has significantly increased prevalence [1], and affects more than 3% of the population in many countries [2–4]. Due to its detrimental effect on the quality of life (QoL) [5] and higher risk of thromboembolic stroke and cardiovascular diseases (CVD) [6, 7], together with its complex interplay with clinical conditions, the hospitalization rate of CVD or non-CVD among AF patients is considerably higher than that of patients without AF [8]. The medical resource consumption and medical expenditure related to AF hospitalization are enormous and continue to increase. A recent Australian study showed that from 1993 to 2013, the number of hospitalized AF patients increased by 295%, which significantly exceeds the increase of myocardial infarction (MI; increased by 73%) and heart failure (HF; increased by 39%) over the same period [9]. Furthermore, Korean National Health Insurance Service (NHIS) database demonstrated that the overall cost of AF hospitalization-related medical care still grows exponentially (from €68.4 million in 2006 to €388.4 million in 2015; relative increase, 468%) even after the adjustment of inflation, which is equivalent to 0.78% of the Korean NHIS total expenditure[10].

According to the findings from the China National Stroke Screening and Prevention Project, the estimated overall prevalence of AF among Chinese adults aged ≥ 40 years in 2014–2015 was 2.31% [11], which is projected to increase further with the economic growth, population aging, and detection tool improvement. Considering that the increasing AF prevalence imposes a significant challenge for the constrained medical resources and brings high financial burden to patients, it is important to understand the available information on hospitalization among AF patients. Nevertheless, little is known about AF-related hospitalization in China. Therefore, this study was designed to analyze data from the China Atrial Fibrillation Registry (China-AF) to examine the incidence, main causes, and risk factors of hospitalization in an unselected cohort of AF patients. Such information is crucial in enabling the development of effective interventions to reduce the overall medical and economic burden in this population.

## Methods

### Study participants

Study participants were from the China-AF study recruited between August 2011 and December 2017. The China-AF is a prospective, multicenter, and hospital-based ongoing registry study. Thirty-one tertiary and non-tertiary hospitals in Beijing providing a clinical service of AF management participated in this registry study [12]. Adult outpatients or inpatients (≥ 18 years old) with a diagnosis of AF were included in the China-AF registry. For the present study, patients were excluded if they met any of the following criteria: (1) transient and reversible AF (e.g. caused by cardiothoracic surgery, hyperthyroidism, and binge drinking), (2) suffering from other serious diseases with a life expectancy < 1 year, (3) diagnosis of rheumatic mitral stenosis or having mitral valve prostheses, (4) HF-related New York Heart Association (NYHA) classification of IV or AF-related European Heart Rhythm Association (EHRA) symptoms classification of IV, (5) follow-up periods less than 6 months.

The study was approved by the Ethics Committee on Human Research in Beijing Anzhen Hospital, the Capital Medical University. All participants provided written informed consent.

### Data collection

Each patient’s demographic data such as age, sex, education level, and health insurance status were collected. AF-specific variables included AF type, duration, the severity of symptoms, and prior treatment. The cardiovascular risk factors were smoking, drinking, and obesity. Current or preexisting comorbid conditions consisted of hypertension (HTN), coronary artery disease (CAD, any history of MI, percutaneous coronary intervention or coronary artery bypass grafting), HF, cardiomyopathy, ischemic stroke/transient ischemic attack (TIA), bleeding, chronic obstructive pulmonary disease (COPD), gastrointestinal disorder, renal dysfunction, hyperthyroidism/hypothyroidism, diabetes mellitus (DM), and hyperlipidemia. The vital signs, laboratory tests, imaging examinations of patients were recorded. Medications of rhythm control agents, rate control agents, antithrombotic drugs, angiotensin-converting enzyme inhibitors (ACEIs)/angiotensin II receptor blockers (ARBs), and cholesterol-lowering agents were collected.

### Outcomes

The primary outcome was all-cause hospitalization during follow-up. The secondary outcomes were cause-specific hospitalization and all-cause death. Patients enrolled were followed up every 6 months either by outpatient clinic visit or by telephone interview. The main cause for hospitalization was determined by the site investigators according to the discharge diagnosis, which was broadly classified into AF, other CVD (CVD excluding AF), and non-CVD. Incidence rates for all-cause and cause-specific hospitalization were calculated as the ratio of the number of hospitalizations to the total follow-up person-time using a unit of per 100 patient-years. The index hospitalization of inpatients recruited in China-AF study were not counted. Patients with recurring multiple hospitalizations during follow-up were counted multiple times.

### Statistical analysis

Patients enrolled in this study were stratified by age (< 65 years; 65–74 years; ≥ 75 years). Continuous variables were presented as mean (standard deviation, SD) and categorical variables were shown as counts (proportions). The comparison of variables of patients in different age groups was analyzed using one-way analysis of variance (ANOVA) (for continuous variables), and chi-square tests (for categorical variables). Hospitalization rate (per 100 patient-years) was expressed by age and sex. Kaplan–Meier curve was used to describe the number of patients at risk, those free of hospitalization, and time to the first all-cause or first cause-specific hospitalization. The Fine-Gray modeling was conducted with (a) the first all-cause hospitalization, (b) the first AF hospitalization, and (c) the first other CVD hospitalization as the dependent variable, and death as competing risks, to evaluate factors that are significantly associated with all-cause and cause-specific hospitalizations. All baseline variables were included in the Fine-Gray modeling. Hazard ratios (HRs) [95% confidence interval (CI)] and P-values were presented. P-value < 0.05 was considered statistically significant. All analyses were performed using SAS statistical software version 9.4 (SAS Institute Inc., Cary, NC).

## Results

### Patient baseline characteristics

A total number of 23,108 patients from 31 sites from August 2011 to December 2017 were enrolled in the China-AF study. After applying the exclusion criteria, 20,172 AF patients were enrolled in this study (a mean age of 64.1 ± 12.0 years; 38.2% female). Their baseline characteristics stratified by age are presented in Table 1. The majority of patients (95.3%) were fully or partially covered by health insurance. More than 1/3 of patients in each age group have at least earned high school diplomas. Patients aged ≥ 75 years had a higher proportion of being female, having persistent AF, having EHRA class of III (AF-related symptoms), having current or preexisting comorbid conditions, including CVD, DM, COPD, and renal dysfunction, using ventricular rate control drugs, antiplatelet agents, ACEIs/ARBs and statins compared to patients in other age groups (all P < 0.01). Patients aged < 65 years tended to have more paroxysmal AF, more cardiovascular risk factors (47.7% overweight, 24.6% obese, 23.2% smoking and 28.3% drinking), a higher probability of receiving the radiofrequency catheter ablation (RFCA), and a more frequent use of antiarrhythmic drugs and direct oral anticoagulants (DOACs) (P < 0.01). There was no significant difference in the proportion of patients with AF duration ≥ 1 year in each age group.

**Table 1:** Baseline characteristics of the patients stratified by age

Characteristics	Total (N = 20,172)	< 65 (N = 9977)	65–74 (N = 5875)	≥ 75 (N = 4320)	P value
Female, n (%)	7707 (38.2)	2866 (28.7)	2740 (46.6)	2101 (48.6)	< 0.01
High school or above, n (%)	6137 (34.1)	3266 (36.4)	1643 (31.2)	1228 (33.0)	< 0.01
Health insurance, n (%)					
No insurance reimbursement	917 (4.7)	550 (5.8)	256 (4.5)	111 (2.6)	< 0.01
Partial insurance reimbursement	16,670 (85.6)	8505 (89.1)	4875 (85.9)	3290 (77.3)	< 0.01
Full insurance reimbursement	1886 (9.7)	487 (5.1)	546 (9.6)	853 (20.1)	< 0.01
BMI, n (%)					
Normal (< 24 kg/m^2^)	6310 (33.2)	2646 (27.7)	1918 (34.5)	1746 (44.7)	< 0.01
Overweight (24–28 kg/m^2^)	8668 (45.6)	4559 (47.7)	2509 (45.1)	1600 (41.0)	< 0.01
Obese (BMI ≥ 28 kg/m^2^)	4036 (21.2)	2344 (24.6)	1131 (20.4)	561 (14.4)	< 0.01
Smoking, n (%)	3170 (15.7)	2317 (23.2)	601 (10.2)	252 (5.8)	< 0.01
Drinking, n (%)	3881 (19.2)	2819 (28.3)	734 (12.5)	328 (7.6)	< 0.01
AF type, n (%)					
Newly diagnosed	1205 (6.0)	473 (4.8)	332 (5.7)	400 (9.3)	< 0.01
Paroxysmal AF	11,692 (58.1)	6038 (60.7)	3465 (59.1)	2189 (50.8)	< 0.01
Persistent AF	7229 (35.9)	3434 (34.5)	2071 (35.3)	1724 (40.0)	< 0.01
AF duration ≥ one year, n (%)	12,236 (60.7)	6015 (60.3)	3626 (61.7)	2595 (60.1)	0.14
EHRA score, n (%)					
I	1655 (9.3)	796 (8.9)	478 (9.2)	381 (10.0)	< 0.01
II	11,520 (64.4)	5952 (66.9)	3265 (63.2)	2303 (60.4)	< 0.01
III	4705 (26.3)	2154 (24.2)	1423 (27.6)	1128 (29.6)	< 0.01
Hypertension, n (%)	13,393 (66.4)	5587 (56.0)	4365 (74.3)	3441 (79.7)	< 0.01
Heart failure, n (%)					
NYHA I	3946 (53.8)	1945 (67.4)	1208 (53.9)	793 (36.0)	< 0.01
NYHA II	2431 (33.2)	735 (25.5)	767 (34.2)	929 (42.1)	< 0.01
NYHA III	957 (13.1)	206 (7.1)	267 (11.9)	484 (21.9)	< 0.01
Established CAD, n (%)	3020 (15.0)	937 (9.4)	1050 (17.9)	1033 (24.0)	< 0.01
Cardiomyopathy, n (%)	365 (1.8)	233 (2.3)	88 (1.5)	44 (1.0)	< 0.01
Ischemic stroke/TIA, n (%)	2884 (14.3)	914 (9.2)	982 (16.7)	988 (22.9)	< 0.01
Haemorrhagic stroke, n (%)	225 (1.1)	91 (0.9)	60 (1.0)	74 (1.7)	< 0.01
Diabetes mellitus, n (%)	4702 (23.3)	1909 (19.1)	1539 (26.2)	1254 (29.0)	< 0.01
Hyperlipidemia, n (%)	9785 (48.5)	5110 (51.2)	2816 (47.9)	1859 (43.0)	< 0.01
Chronic obstructive pulmonary disease, n (%)	169 (0.8)	35 (0.4)	54 (0.9)	80 (1.9)	< 0.01
Gastrointestinal disorder, n (%)	633 (3.1)	299 (3.0)	189 (3.2)	145 (3.4)	0.48
Hyperthyroidism/Hypothyroidism, n (%)	994 (4.9)	448 (4.5)	323 (5.5)	223 (5.2)	0.01
Renal dysfunction, n (%)	566 (3.5)	85 (1.1)	137 (3.0)	344 (10.2)	< 0.01
CHA2DS2-VASc score ≥ 2, n (%)	13,424 (75.4)	3790 (49.8)	5314 (90.5)	4320 (100.0)	< 0.01
Antiarrhythmic drugs, n (%)	7330 (36.3)	4454 (44.6)	1969 (33.5)	907 (21.0)	< 0.01
Ventricular rate control, n (%)	9575 (47.5)	4096 (41.1)	2951 (50.2)	2528 (58.5)	< 0.01
Warfarin, n (%)	9449 (46.8)	4762 (47.7)	2941 (50.1)	1746 (40.4)	< 0.01
DOACs, n (%)	3268 (16.2)	1975 (19.8)	867 (14.8)	426 (9.9)	< 0.01
Aspirin/ Clopidogrel, n (%)	4894 (24.3)	1879 (18.8)	1478 (25.2)	1537 (35.6)	< 0.01
ACEIs/ARBs, n (%)	6828 (33.9)	2690 (27.0)	2265 (38.6)	1873 (43.4)	< 0.01
Statin, n (%)	7461 (37.0)	2987 (29.9)	2522 (42.9)	1952 (45.2)	< 0.01
History of RFCA, n (%)	8801 (43.6)	5595 (56.1)	2355 (40.1)	851 (19.7)	< 0.01
Echocardiogram parameters					
LAD, mm, (SD)	40.4 ± 6.5	39.8 ± 6.3	40.5 ± 6.4	41.7 ± 7.0	< 0.01
IVS, mm, (SD)	9.8 ± 1.7	9.7 ± 1.8	9.8 ± 1.7	9.9 ± 1.8	< 0.01
LVEDD, mm, (SD)	48.3 ± 5.6	48.8 ± 5.5	47.8 ± 5.5	47.8 ± 5.9	< 0.01
LVEF, %, (SD)	62.9 ± 8.3	62.7 ± 8.2	63.5 ± 8.2	62.6 ± 8.9	< 0.01

BMI, body mass index; AF, atrial fibrillation; CAD, coronary artery disease; TIA, transient ischemic attack; DOAC, direct oral anticoagulants; ACEIs, angiotensin-converting enzyme inhibitors; ARBs, angiotensin II receptor blockers; RFCA, radiofrequency catheter ablation; LAD, left atrial diameter; LVEF, left ventricular ejection fraction; IVS, interventricular septum; LVEDD, left ventricular end-diastolic dimension. eGFR (ml/min·1.73m2) = 186 × Scr-1.154 × ge-0.203 .742 (if female) 1.233 (if Chinese). Renal dysfunction was defined as eGFR < 60 ml/min·1.73m^2^. According to the proportion of medical insurance company payment, health insurance was divided into three levels: full, partial, and no insurance reimbursement

### Rate and causes of hospitalization

**Fig. 1:**
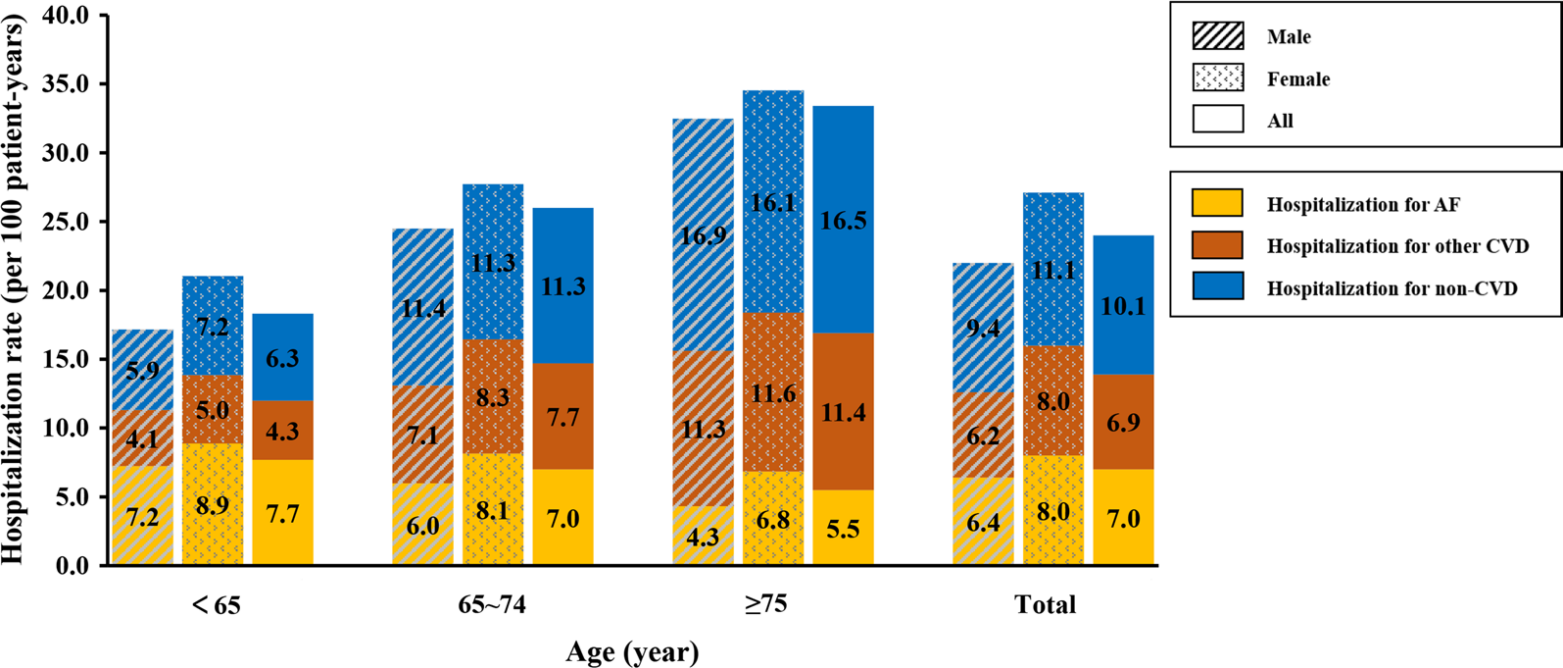
Hospitalization rate (per 100 patient-years) for different causes across age and gender groups. AF, atrial fibrillation; CVD, cardiovascular diseases

**Fig. 2:**
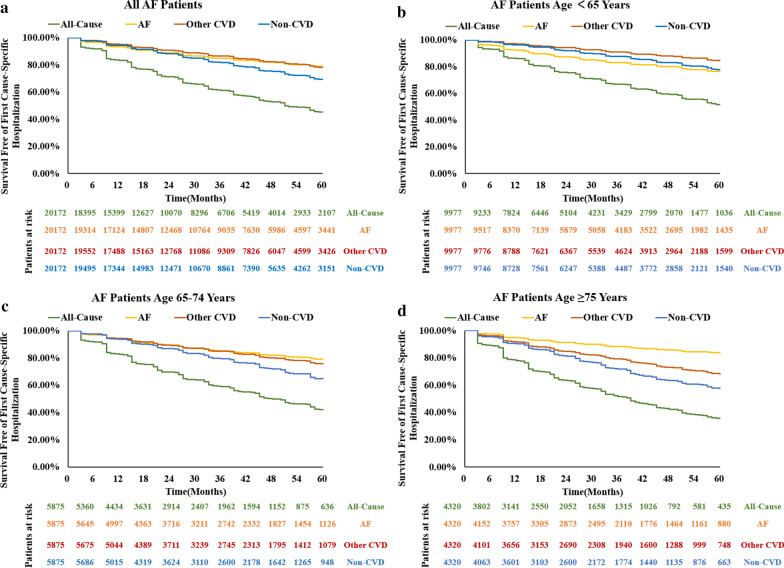
Survival free of first all-cause and first specific-cause hospitalization in different age groups. **a** the entire cohort; **b** patients age < 65 years; **c** patients age 65–74 years; **d** patients age ≥ 75 years. AF, atrial fibrillation; other CVD, cardiovascular diseases excluding AF; non-CVD, non-cardiovascular diseases

Over a mean follow-up of 37.3 ± 20.4 months, a total number of 14,828 hospitalizations, 1,277 deaths were identified, 7,512 patients (37.2%) had at least one hospitalization. The overall incidence of all-cause hospitalization and all-cause death were 24.0 per 100 patient-years and 2.1 per 100 patient-years, respectively. Cause-specific hospitalization rates of AF patients by age groups and sex were shown in Fig. 1. Hospitalization rates of patients aged < 65, 65–74, and ≥ 75 years were 18.3 per 100 patient-years, 26.0 per 100 patient-years, and 33.5 per 100 patient-years, respectively. Hospitalizations occurred most in female patients aged ≥ 75 years and were more common among females of all age groups compared to their male counterparts. Among patients aged < 65 years, hospitalizations attributed to AF accounted for the largest proportion, reaching 42.1% of the all-cause hospitalizations, while patients aged 65–74 and ≥ 75 years were predominantly hospitalized for non-CVD (43.6% and 49.3% respectively). Kaplan–Meier curves of the first hospitalization for all-cause, AF, other CVD, or non-CVD causes were displayed in Fig. 2.

### Factors associated with first all-cause hospitalization

The multivariate analysis identified several risk factors of first all-cause hospitalizations in AF patients (Table 2). Elderly patients were more likely to have all-cause hospitalization. As indicated by our results, compared with patients in the < 65 age group, patients in the 65–74 age group (HR 1.18, 95% CI 1.12–1.25, P < 0.01) and in the ≥ 75 age group (HR 1.34, 95% CI 1.25–1.43, P < 0.01) were more likely to experience hospitalization. EHRA II (HR 1.13, 95% CI 1.03–1.24, P = 0.01) and EHRA III (HR 1.18, 95% CI 1.06–1.31, P < 0.01) showed a significantly predictive role for all-cause hospitalization. Paroxysmal AF, AF duration ≥ 1 year, and left atrial enlargement were highly associated with risks of all-cause hospitalization. In addition, HTN (HR 1.08, 95% CI 1.02–1.15), HF (HR 1.10, 95% CI 1.02–1.18), CAD (HR 1.24, 95% CI 1.17–1.33), and ischemic stroke/TIA (HR 1.22, 95% CI 1.15–1.30) also contributed to the increased risk of all-cause hospitalization. For AF patients comorbid with HF, there was no difference in all-cause hospitalizations by left ventricular ejection fraction classifications (see Additional file 1: Table 1). Furthermore, non-cardiovascular comorbidities including DM (HR 1.14, 95% CI 1.08–1.20), COPD (HR 1.28, 95% CI 1.02–1.62), gastrointestinal disorder (HR 1.37, 95% CI 1.21–1.55), hyperthyroidism/hypothyroidism (HR 1.25, 95% CI 1.13–1.39), and renal dysfunction (HR 1.24, 95% CI 1.09–1.42) were associated with a higher all-cause hospitalization rate during follow-up. Of note, the use of ACEIs/ARB and history of RFCA were identified as protective factors of all-cause hospitalization. The risk factors of all-cause death in AF patients are shown in Additional file 2: Table 2.

**Table 2:** Factors associated with all-cause hospitalization

Factors	Multivariate analysis	
	HR (95%CI)	P value
*Age (years)*		
< 65	Ref	–
65–74	1.18 (1.12, 1.25)	< 0.01
≥ 75	1.34 (1.25, 1.43)	< 0.01
Female	1.06 (1.01, 1.12)	0.03
*Health insurance*		
No insurance reimbursement	Ref	–
Partial insurance reimbursement	1.12 (1.00, 1.25)	0.05
Full insurance reimbursement	1.06 (0.93, 1.21)	0.40
*AF type*		
Newly diagnosed	Ref	–
Paroxysmal AF	1.17 (1.05, 1.29)	< 0.01
Persistent AF	1.11 (0.99, 1.23)	0.07
AF duration ≥ 1 year	1.12 (1.06, 1.18)	< 0.01
*EHRA score*		
I	Ref	–
II	1.13 (1.03, 1.24)	0.01
III	1.18 (1.06, 1.31)	< 0.01
Hypertension	1.08 (1.02, 1.15)	< 0.01
Heart failure	1.10 (1.02, 1.18)	< 0.01
Established CAD	1.24 (1.17, 1.33)	< 0.01
Ischemic stroke/TIA	1.22 (1.15, 1.30)	< 0.01
Diabetes mellitus	1.14 (1.08, 1.20)	< 0.01
Chronic obstructive pulmonary disease	1.28 (1.02, 1.62)	0.03
Gastrointestinal disorder	1.37 (1.21, 1.55)	< 0.01
Hyperthyroidism/Hypothyroidism	1.25 (1.13, 1.39)	< 0.01
Renal dysfunction	1.24 (1.09, 1.42)	< 0.01
Ventricular rate control	1.06 (1.01, 1.12)	0.01
ACEIs/ARBs	0.94 (0.90, 1.00)	0.03
History of RFCA	0.92 (0.87, 0.98)	0.02
LAD, (per 1 mm increase)	1.01 (1.00, 1.01)	< 0.01

AF, atrial fibrillation; CAD, coronary artery disease; TIA, transient ischemic attack; ACEIs, angiotensin-converting enzyme inhibitors; ARBs, angiotensin II receptor blockers; RFCA, radiofrequency catheter ablation; LAD, left atrial diameter; HR, hazard ratio; 95% CI, 95% confidence interval

### Factors associated with first cause-specific hospitalization

We found that the major predictors of AF hospitalization were severe symptoms (EHRA II, HR 1.32, 95% CI 1.11–1.58, P < 0.01; EHRA III, HR 1.32, 95% CI 1.08–1.61, P < 0.01) and antiarrhythmic drugs (HR 1.38, 95% CI 1.26–1.50, P < 0.01) (Table 3). For other CVD hospitalization, the predictor was complicating CVD (Table 4). Additionally, we observed that RFAC was associated with a decrease of AF-related hospitalization (11%) (HR 0.89, 95% CI 0.81–0.99, P = 0.04) and subsequent CVD hospitalization (14%) (HR 0.86, 95% CI 0.77–0.96, P < 0.01).

**Table 3:** Factors associated with AF hospitalization

Factors	Multivariate analysis	
	HR (95%CI)	P value
*Age (years)*		
< 65	Ref	–
65–74	0.83 (0.76, 0.90)	< 0.01
≥ 75	0.64 (0.57, 0.72)	< 0.01
Female	1.18 (1.09, 1.29)	< 0.01
High school or above	1.11 (1.02, 1.20)	0.01
AF duration ≥ 1 year	1.29 (1.18. 1.40)	< 0.01
*EHRA score*		
I	Ref	–
II	1.32 (1.11, 1.58)	< 0.01
III	1.32 (1.08, 1.61)	< 0.01
Established CAD	1.19 (1.07, 1.33)	< 0.01
Antiarrhythmic drugs	1.38 (1.26, 1.50)	< 0.01
History of RFCA	0.89 (0.81, 0.99)	0.04

AF, atrial fibrillation; CAD, coronary artery disease; RFCA, radiofrequency catheter ablation; HR, hazard ratio; 95% CI, 95% confidence interval

**Table 4:** Factors associated with other CVD hospitalization

Factors	Multivariate analysis	
	HR (95%CI)	P value
*Age (years)*		
< 65	Ref	–
65–74	1.39 (1.26, 1.54)	< 0.01
≥ 75	1.63 (1.46, 1.81)	< 0.01
High school or above	0.89 (0.81, 0.98)	0.02
Hypertension	1.20 (1.08, 1.32)	< 0.01
Heart failure	1.42 (1.27, 1.58)	< 0.01
Established CAD	1.42 (1.29, 1.57)	< 0.01
Ischemic stroke/TIA	1.33 (1.21, 1.46)	< 0.01
Diabetes mellitus	1.20 (1.10, 1.30)	< 0.01
Gastrointestinal disorder	1.41 (1.14, 1.74)	< 0.01
Hyperthyroidism/Hypothyroidism	1.23 (1.02, 1.47)	0.03
Renal dysfunction	1.23 (1.04, 1.46)	0.02
Statin	1.16 (1.06, 1.26)	< 0.01
History of RFCA	0.86 (0.77, 0.96)	< 0.01
LAD, (per 1 mm increase)	1.01 (1.01, 1.02)	< 0.01

CAD, coronary artery disease; TIA, transient ischemic attack; RFCA, radiofrequency catheter ablation; LAD, left atrial diameter; HR, hazard ratio; 95% CI, 95% confidence interval

## Discussion

Hospitalization is common in AF patients. In our study, we observed a hospitalization rate of 24.0 per 100 patient-years, and women aged ≥ 75 years had the highest hospitalization rate. With the increase of age, elderly AF patients (≥ 65 years) were more frequently hospitalized for non-CVD reasons. Age, AF types and symptoms, as well as several comorbidities were independent predictors for first all-cause hospitalization.

In China, there was few data focusing on the hospitalization rate in AF patients. The present study is the first study that analyzed the total frequency of hospitalizations to evaluate the incidence rate of hospitalization in AF patients based on real-world data in China. In 2014, the ORBIT-AF study revealed that the rate of all-cause hospitalization in AF patients was as high as 38.8 per 100 patient-years [13]. Furthermore, data from the EORP-AF Pilot registry in European Society of Cardiology nine-member countries, showed that the annual hospitalization rate in AF patients was up to 39.3 per 100 patient-years [14]. According to our study, the all-cause hospitalization rate in AF patients was 24.0 per 100 patient-years. Although the hospitalization rate of AF patients in China maybe not as high as that in western countries, China is facing similar disease and economic burden of hospitalization in AF patients as western countries.

To date, the main causes for hospitalization in AF patients are still highly controversial. The present study demonstrated that non-CVD were most responsible for hospitalizations in AF patients, which is similar to numerous previous researches [8, 15, 16]. However, some studies such as ROCKET AF study [17], ORBIT-AF I and II registry [13, 18] support opposite views to our results, showing that AF patients are hospitalized mainly for CVD. In patients aged < 65 years, due to few complicating diseases and high requirements for QoL, most young people may be hospitalized for relieving AF-caused symptoms or seeking catheter ablation to maintain sinus rhythm. The triggering or exacerbating effects of other diseases caused by pathophysiological alterations [19–21], old age, and the adverse events following pharmaceutical treatment of AF [22–24], put older and multi-morbid patients at a greater risk of getting adverse outcomes across multiple organs other than AF or cardiac events. Some of the conflicting findings in these studies regarding the main cause for hospitalization in AF patients may be attributable to different clinical setting and patient population from other studies. Therefore, different therapeutic strategies are required for problems presented in different age groups. Symptoms improvements and treatment focusing on AF alone may be more effective in young AF patients, while the concomitant diseases should be paid more attention to in elderly AF patients.

To improve our identification of predictors of hospitalization and focus areas for preventive efforts and intervention, we examined the association between patient baseline characteristics and hospitalization rate in AF patients. The risk of hospitalization increased substantially with the increase of age, suggesting that the burden of hospitalization for AF patients is anticipated to increase dramatically with the aging population. Of note, in the multivariate model, female was an independent risk factor for hospitalization in patients with AF. Among all age groups, the all-cause hospitalization rate of female AF patients was higher than that of male AF patients. A previous study has pointed out that women with AF tend to be more symptomatic and experience worse QoL than men [25]. However, a meta-analysis of 17 articles provides an equivocal conclusion on gender differences in hospitalizations [26]. Therefore, more researches should be done to evaluate these gender differences in AF hospitalization.

Consistent with numerous studies [27, 28], patients with worse symptoms by ERHA classification had a higher risk of hospitalization. When we modeled predictors of hospitalization for AF, symptom status was a major driver. Similarly, patients requiring antiarrhythmic drugs were also more likely to experience hospitalization for AF. These results suggested the need to manage patients with symptomatic and/or uncontrolled AF, so as to reduce the incidence of hospitalization. Catheter ablation, a new treatment approach for AF, not only showed a significant association with all-cause hospitalization in our study, but also improved symptoms [29] and reduced subsequent cardiovascular events [30].

Moreover, as indicated by our data, the combination of CVD (such as stroke, HF and CAD) in AF patients could significantly predict subsequent hospitalization, especially non-AF CVD hospitalizations. It is generally known that stroke, HF, and CAD are frequent comorbidities among AF patients. Given the common underlying pathophysiology and similar management focuses [31–33], AF patients are prone to a number of cardiometabolic comorbidities, particularly HF and stroke [34]. AF patients comorbid with CVD also tend to have relapse or progressed AF. Chamberlain et al. [15] found that AF patients complicated with HF experienced an increased risk of hospitalization by up to about 70%, and those complicated with CAD and stroke also had an over 20% increased risk of hospitalization. Furthermore, these CVD themselves alone represent a significant burden of hospitalization. Accordingly, improving the integrated management of CVD and reducing the frequency of recurrent hospitalizations in AF patients complicated with CVD are in an urgent need.

In our cohort, it is worth noting that gastrointestinal disorder patients had the highest risk of all-cause hospitalization, showing an increase of about 37%, followed by COPD and renal dysfunction. In addition to increasing the risk of all-cause hospitalization, gastrointestinal disorder and renal dysfunction also elevated the risk of non-AF CVD hospitalization by 41% and 23% respectively. As has been suggested by a previous study, renal diseases not only induce and aggravate CVD such as hypertension, HF and CAD [35], but also increase the risk of stroke, systemic thromboembolism and bleeding [36, 37]. Gastrointestinal disorders such as gastroesophageal reflux and gastroenteritis may also elevate the risk of bleeding or cardiovascular events due to inflammation, metabolic disorders or treatment [38, 39]. Moreover, renal dysfunction and gastrointestinal disorder could lead to the reduction of the drug compliance of AF patients [40, 41]. Therefore, when managing AF patients complicated with non-CVD, cardiologists could pay more attention to preexisting non-CVD to add more value to the integrated management of such comorbid patients.

Collectively, the majority of comorbidities (CVD/non-CVD) predicted a high prevalence of hospitalization, the care oriented to single disease treatment rather than targeting broad and interconnected care may make interventions and outcomes unsatisfying. Multidisciplinary management of AF and comorbidities is an inexorable trend of medical care.

## Limitations

Firstly, in this study, AF patients were recruited mainly from the urban and suburban areas, which principally mirrors the circumstance of AF patients in relatively developed regions in China, and may be insufficient to represent the whole AF population in China, especially in rural areas. Additional studies should be undertaken to determine whether these observations are generalizable to all AF patients. Secondly, the analysis data were derived from the China-AF study. The causes for the hospitalization proposed by the researchers were relatively broad. The non-AF CVD and non-CVD were not explicitly classified. Therefore, the most important specific diseases that lead to the hospitalization of AF patients are unavailable and need further investigation. Lastly, this study did not collect detailed information about the length of stay and hospitalization cost and could not further analyze the economic burden of hospitalization.

## Conclusions

During follow-up, about one-third of AF patients have had more than one hospitalization in this study. Hospitalizations mainly due to non-CVD events in patients aged ≥ 65 years and AF burden in the patients aged < 65 years. Age, worse AF symptoms and several comorbidities (e.g. HF and COPD) are significant predictors for hospitalization. Throughout AF patients’ entire treatment and follow-up, efforts are needed to reduce hospitalizations, particularly in focusing on symptoms, as well as strengthening the multidisciplinary management of comorbidities.

## Supplementary Information

**Additional file 1.** Hospitalization rate (per 100 patient-years) of AF patients with HF by different classification.

**Additional file 2.** Factors associated with all-cause death.

## Data Availability

The datasets generated and/or analysed during the current study are not publicly available due to the protection of patients’ privacy, but are available from the corresponding author on reasonable request.

## Abbreviations

AF: Atrial Fibrillation
ACEIs: Angiotensin-Converting Enzyme Inhibitors
ARBs: Angiotensin II Receptor Blockers
BMI: Body Mass Index
CAD: Coronary Artery Disease
CHA_2_DS_2_-VASc: Congestive heart failure, Hypertension, Age ≥ 75 years, Diabetes, Stroke, Vascular diseases, Age 65–74 years, Sex category
China-AF: Chinese Atrial Fibrillation Registry
CI: Confidence Interval
COPD: Chronic Obstructive Pulmonary Disease
CVD: Cardiovascular Diseases
DM: Diabetes Mellitus
DOACs: Direct Oral Anticoagulants
EHRA: European Heart Rhythm Association
HF: Heart Failure
HR: Hazard Ratio
HTN: Hypertension
LAD: Left Atrial Diameter
LVEF: Left Ventricular Ejection Fraction
MI: Myocardial Infarction
NHIS: National Health Insurance Service
NYHA: New York Heart Association
QoL: Quality of Life
RFCA: Radiofrequency Catheter Ablation
TIA: Transient Ischemic Attack
