# Supplementary material for: Incidence and predictors of hospitalization in patients with atrial fibrillation: results from the Chinese atrial fibrillation registry study
Source: BMC Cardiovasc Disord. 2021 Mar 19;21:146. doi: 10.1186/s12872-021-01951-5 (PMC7980549; doi:10.1186/s12872-021-01951-5)
Supplement: Supplementary file 1 — Additional file 1. Hospitalization rate (per 100 patient-years) of AF patients with HF by different classification. [file 12872_2021_1951_MOESM1_ESM.docx]

**Supplementary Appendix**

**Table 1. Hospitalization rate (per 100 patient-years) of AF patients with HF by different classification**

|  | **HFrEF**  **(n=317)** | **HFmrEF**  **(n=349)** | **HFpEF**  **(n=2600)** | **P^1^ value** | **P^2^ value** |
| --- | --- | --- | --- | --- | --- |
| All-cause | 20.3 | 21.5 | 19.5 | 0.63 | 0.65 |
| AF-related | 4.1 | 3.9 | 4.4 | 0.82 | 0.69 |
| Cardiovascular -related | 8.1 | 10.7 | 7.6 | 0.10 | 0.61 |

P^1^: Results of comparison between HFrEF group and HFmrEF group.

P^2^: Results of comparison between HFrEF group and HFpEF group.
