# Supplementary material for: Incidence and predictors of hospitalization in patients with atrial fibrillation: results from the Chinese atrial fibrillation registry study
Source: BMC Cardiovasc Disord. 2021 Mar 19;21:146. doi: 10.1186/s12872-021-01951-5 (PMC7980549; doi:10.1186/s12872-021-01951-5)
Supplement: Supplementary file 2 — Additional file 2. Factors associated with all-cause death. [file 12872_2021_1951_MOESM2_ESM.docx]

**Supplementary Appendix**

**Table 2. Factors associated with all-cause death**

| **Factors** | **Multivariate analysis** | |
| --- | --- | --- |
|  | **HR (95%CI)** | **P value** |
| Age (years) |  |  |
| < 65 | Ref | - |
| 65-74 | 2.19 (1.82, 2.64) | < 0.01 |
| ≥75 | 4.86 (4.06, 5.82) | < 0.01 |
| High school or above | 0.82 (0.71, 0.94) | < 0.01 |
| BMI, n (%) |  |  |
| Normal (<24 kg/m²) | Ref | - |
| Overweight (24-28 kg/m²) | 0.81 (0.71, 0.93) | 0.01 |
| Obese (BMI ≥28kg/m²) | 0.72 (0.60, 0.86) | 0.02 |
| Smoking | 1.47 (1.22, 1.75) | < 0.01 |
| Heart failure | 1.90 (1.62, 2.23) | < 0.01 |
| Established CAD | 1.18 (1.03, 1.35) | 0.01 |
| Ischemic stroke/TIA | 1.54 (1.36, 1.75) | < 0.01 |
| Diabetes mellitus | 1.22 (1.08, 1.38) | < 0.01 |
| Chronic obstructive pulmonary disease | 1.72 (1.18, 2.51) | < 0.01 |
| Cardiomyopathy | 1.78 (1.32, 2.40) | < 0.01 |
| Renal dysfunction | 1.72 (1.44, 2.04) | < 0.01 |
| Warfarin | 0.66 (0.56, 0.77) | < 0.01 |
| DOACs | 0.57 (0.38, 0.85) | < 0.01 |
| History of RFCA | 0.63 (0.51, 0.77) | < 0.01 |
| LAD, (per 1mm increase) | 1.02 (1.01, 1.03) | < 0.01 |
| LVEF, (per 1% increase) | 0.99 (0.98, 0.99) | < 0.01 |

BMI, body mass index; CAD, coronary artery disease; TIA, transient ischemic attack; DOAC, direct oral anticoagulants; RFCA, radiofrequency catheter ablation; LAD, left atrial diameter; LVEF, left ventricular ejection fraction. HR, hazard ratio; 95% CI, 95% confidence interval.
